# Gut microbiota-derived butyrate contributes to baicalin-induced attenuation of hypertensive vascular remodeling via adventitial immunity

**DOI:** 10.3389/fphar.2026.1835174

**Published:** 2026-07-03

**Authors:** Qiurong Xie, Yanyan Yang, Jiekun Lin, Yuehong Ye, Jiyan Lin, Meizhu Wu, Zhi Guo, Aling Shen, Weiquan Zeng, Jun Peng

**Affiliations:** 1 Department of Scientific Research and Teaching, Affiliated Rehabilitation Hospital of Fujian University of Traditional Chinese Medicine, Fuzhou, Fujian, China; 2 Innovation and Transformation Center, Fujian University of Traditional Chinese Medicine, Fuzhou, Fujian, China; 3 Fujian Key Laboratory of Integrative Medicine on Geriatrics, Fujian University of Traditional Chinese Medicine, Fuzhou, Fujian, China; 4 Fujian Collaborative Innovation Center for Integrative Medicine in Prevention and Treatment of Major Chronic Cardiovascular Diseases, Fuzhou, Fujian, China; 5 Academy of Integrative Medicine, College of Integrative Medicine, Fujian University of Traditional Chinese Medicine, Fuzhou, Fujian, China; 6 College of Pharmacy, Fujian University of Traditional Chinese Medicine, Fuzhou, Fujian, China; 7 Department of Orthopaedics, Affiliated Rehabilitation Hospital of Fujian University of Traditional Chinese Medicine, Fuzhou, Fujian, China

**Keywords:** adventitial immunity, baicalin, gut microbiota, hypertension, regulatory T cells, short-chain fatty acids, vascular remodeling

## Abstract

**Background:**

Baicalin shows potent vasculoprotective effects against hypertension despite poor oral bioavailability. We investigated whether gut microbiota modulation contributes to the systemic vasculoprotective effects of orally administered baicalin.

**Methods:**

We utilized an Angiotensin II-induced hypertensive mouse model, employing broad-spectrum antibiotics, 16S rRNA sequencing, metabolomics, and *in vitro* co-culture assays to map the gut-immune-vascular axis.

**Results:**

Oral Baicalin significantly attenuated Ang II-induced blood pressure elevation and improved the intestinal barrier integrity. Antibiotic-induced microbiota depletion substantially weakened these protective effects, supporting a major microbiota contribution under the present experimental conditions. Baicalin reshaped the gut microbial community, enriched SCFA-supporting taxa, and restored a putative butyrate-associated microbial signature, enriching *Akkermansia* and *Lactobacillus* accompanied by increased cecal butyrate levels. Exogenous sodium butyrate recapitulated several major protective features of Baicalin treatment, including expansion of Foxp3+ regulatory T cells in mesenteric lymph nodes. These immune changes were accompanied by increased Foxp3+ regulatory immune-cell accumulation in the aortic adventitia. *In vitro*, butyrate-licensed Tregs suppressed Ang II-induced vascular smooth muscle cell (VSMC) proliferative responses, at least partly through IL-10-mediated inhibition of MAPK/ERK signalling.

**Conclusion:**

Baicalin alleviates Ang II-associated vascular remodelling, at least in part, by reprogramming gut microbial ecology, increasing luminal butyrate availability, promoting regulatory immune responses, and suppressing VSMC proliferative signalling.

## 1 Introduction

Hypertension drives global cardiovascular mortality, primarily precipitated by maladaptive vascular remodelling and endothelial dysfunction (Mancia et al., 2023; Faraci, 2011). Current pharmacological strategies effectively modulate the haemodynamic load. However, clinical cohorts frequently exhibit resistant hypertension. Pathological structural changes, such as medial thickening, fibrosis, and vascular smooth muscle cell (VSMC) hyperplasia, persist despite optimised blood pressure control (Judd and Calhoun, 2014). This residual risk indicates that conventional vasodilators fail to intercept the immunometabolic aetiology of vascular pathology (Dasinger et al., 2025). Hypertension is not only a mechanical disorder but also manifests as a systemic inflammatory syndrome. Disruption of T-cell homeostasis orchestrates the transition from transient vasoconstriction to permanent arterial damage. Specifically, pro-inflammatory Th17 cells expand at the expense of immunosuppressive regulatory T cells (Tregs) (Ding et al., 2022; Meng et al., 2016). Recalibrating this immune equilibrium offers a targeted approach for mitigating end-organ damage.

The intestine serves as the primary immunological checkpoint governing systemic tone. A functional gut-immune-vascular axis requires an intact epithelial barrier coupled with the metabolic output of the commensal microbiota (O’Donnell et al., 2023). Dysbiosis, defined by contracted microbial diversity and depleted short-chain fatty acid (SCFA) producers, compromises this epithelial seal. Luminal lipopolysaccharide (LPS) subsequently translocate into the systemic circulation (Snelson et al., 2024). This leaky gut phenotype is a chronic inflammatory stimulus. Gut-associated lymphoid tissue (GALT) skews naive T cell differentiation toward pathogenic Th17 lineages (Dinakis et al., 2024). Conversely, microbial metabolites such as butyrate function as epigenetic regulators. By inhibiting histone deacetylases, butyrate promotes Foxp3 expression to biologically “license” Tregs with suppressive capacity (Furusawa et al., 2013; Mann et al., 2024). The correlation between gut dysbiosis and hypertension is well documented. However, the precise mechanism by which specific microbial metabolites pharmacologically arrest distal vascular remodelling remains unclear (Avram et al., 2025).

Baicalin, a bioactive flavonoid extracted from Scutellaria baicalensis, exerts robust antihypertensive and vasculoprotective effects in rats. However, its clinical application encounters a severe pharmacokinetic paradox (Hu et al., 2025). Baicalin has negligible oral bioavailability and undergoes rapid hydrolysis within the gastrointestinal tract, despite its potent systemic efficacy (Fan et al., 2025). Such discordance raises the possibility that the gut microbiota may represent an important pharmacological interface contributing to the systemic actions of orally administered baicalin, rather than acting merely as a passive absorption barrier (Muttiah and Hanafiah, 2025). Baicalin structurally modulates gut flora (Wu et al., 2019). Whether this ecological restructuring translates into a verifiable metabolic signal instructing the adaptive immune system to protect the vasculature remains unclear. The specific causality connecting baicalin-induced metabolic reprogramming to the systemic mobilisation and adventitial accumulation of Tregs lacks definitive proof.

We therefore investigated whether oral baicalin attenuates Ang II-induced hypertensive vascular remodeling through a gut microbiota–SCFA–regulatory immunity axis. Using antibiotic-mediated microbiota depletion, 16S rRNA sequencing, untargeted metabolomics, targeted SCFA quantification, flow cytometry, vascular histology, immunofluorescence, and *in vitro* co-culture assays, we examined whether baicalin-associated microbial remodeling is linked to luminal butyrate availability, MLN regulatory immune priming, adventitial Foxp3+ regulatory immune-cell accumulation, and VSMC proliferative signaling.

## 2 Materials and methods

### 2.1 Animals and angiotensin II-induced hypertension model

Male C57BL/6 J mice (8–10 weeks old, 20–25 g; Vital River Laboratory Animal Technology, Beijing, China) were housed in specific pathogen-free (SPF) facilities. Environmental parameters were strictly controlled (22 °C ± 2 °C, 55% ± 5% humidity, 12-h light/dark cycle) with *ad libitum* access to standard chow and water. Following a one-week acclimatisation period, subcutaneous implantation of osmotic minipumps (Alzet Model 2004, DURECT Corp.) induced hypertension (Bartolomaeus et al., 2019). Under 2% isoflurane anaesthesia, dorsal implantation delivered Ang II (Sigma-Aldrich) at a continuous rate of 500 ng/kg/min for 28 days. A CODA™ noninvasive tail-cuff plethysmography system (Kent Scientific, Torrington, CT, United States) continuously monitored systolic blood pressure (SBP), diastolic blood pressure (DBP), and mean arterial pressure (MAP) weekly. To eliminate stress-induced artefacts, the mice underwent a 15-min daily training regimen on a 37 °C heating pad for three consecutive days prior to baseline quantification. All protocols were approved by the Institutional Animal Care and Use Committee of the Fujian University of Traditional Chinese Medicine (no. FUTCM IACUC 2025339).

### 2.2 Experimental design and pharmacological interventions

Mice were initially randomised into six cohorts (n = 8–10/group). Following standard exclusion criteria for surgical non-responders and applying the ROUT method (Q = 1%) to remove technical outliers, a standardised sample size of n = 7 per group was strictly maintained for all downstream functional, histological, and molecular analyses: (1) Sham (saline infusion); (2) Ang II (model); (3) Ang II + Baicalin (50 mg/kg/day, intragastric gavage); (4) Ang II + Baicalein (50 mg/kg/day, intraperitoneal injection); (5) Ang II + Baicalin + Antibiotics (broad-spectrum cocktail); and (6) Ang II + Sodium Butyrate (NaB, 200 mg/kg/day, intragastric gavage). MedChemExpress supplied Baicalin (≥98% purity) and Baicalein.

The selected doses for Baicalin (50 mg/kg/day) and Sodium Butyrate (200 mg/kg/day) were based on previously established pharmacological efficacy profiles in murine models, representing standard therapeutic ranges that effectively modulate gut microbiota and elicit systemic cardiovascular protection without inducing overt toxicity (Ding et al., 2019; Chen et al., 2019; Zhao et al., 2020; Xiong et al., 2022; Zhang et al., 2023). Importantly, the intraperitoneal (i.p.) Baicalein group served as an essential route-of-administration control. Because oral Baicalin has limited systemic bioavailability and is extensively metabolized within the gastrointestinal tract, comparing oral baicalin against systemic (i.p.) delivery of its aglycone baicalein allowed us to evaluate whether the protective phenotype was preferentially associated with enteral gut-microbiota interactions rather than systemic exposure alone.

To achieve microbiota depletion, mice consumed an antibiotic cocktail—Ampicillin (1 g/L), Vancomycin (0.5 g/L), Neomycin (1 g/L), and Metronidazole (1 g/L) (Solarbio)—*ad libitum* in drinking water (Shikata et al., 2019). This regimen was initiated 1 week prior to Ang II infusion and persisted continuously, with solutions renewed every 48 h.

### 2.3 Preliminary safety evaluation of selected doses

To preliminarily assess the tolerability of the selected doses, healthy male C57BL/6 J mice were randomly assigned to receive vehicle, Baicalin at 50 mg/kg/day, or Sodium Butyrate (NaB) at 200 mg/kg/day for 28 days (n = 3 per group). Body weight was recorded on days 0, 7, 14, 21, and 28. At the endpoint, heart, liver, and kidney tissues were fixed in 4% paraformaldehyde, embedded in paraffin, sectioned, and subjected to hematoxylin and eosin (H&E) staining to examine major-organ histopathology.

### 2.4 16S rRNA gene sequencing and bioinformatics

Fresh caecal contents were snap-frozen in liquid nitrogen immediately after dissection. Microbial genomic DNA was extracted using the TIANamp Stool DNA Kit (Tiangen) via a rigid lysis protocol. Barcoded primers (338F: 5′-ACT​CCT​ACG​GGA​GGC​AGC​AG-3′; 806R: 5′-GGACTACHVGGGTWTCTAAT-3′) were used to amplify the V3–V4 hypervariable regions of the bacterial 16S rRNA gene. Following purification (AxyPrep DNA Gel Extraction Kit) and quantification (QuantiFluor™-ST), the NEBNext® Ultra™ DNA Library Prep Kit was used to generate sequencing libraries. The Illumina NovaSeq 6000 platform produced 250 bp paired-end reads. The QIIME 2 pipeline (v2022.2) was used to filter and process the raw data (Bolyen et al., 2019).

To identify specific microbial biomarkers, Linear Discriminant Analysis Effect Size (LEfSe) was executed. To ensure high stringency and transparency, the predefined parameters included an alpha value of 0.05 for the factorial Kruskal-Wallis test and a strict Log10 LDA score threshold of >3.0, coupled with FDR correction (*q* < 0.05). The genus-level heatmap displays the top-ranked differentially abundant taxa selected from the predefined LEfSe output rather than manually curated genera. The Chao1 and Shannon indices were used to quantify alpha diversity. Principal Coordinate Analysis (PCoA) operating on Bray-Curtis dissimilarity matrices was used to visualise beta diversity. The complete list of significant genus-level taxa, including LDA scores, raw P-values, and FDR-adjusted q-values, is provided in Supplementary Table S1.

### 2.5 Untargeted metabolomics profiling and targeted SCFA quantification

An LC-MS/MS system was used to map global metabolic shifts within the caecal contents. The raw data underwent rigorous peak alignment and integration. Multivariate statistical frameworks were used to map the metabolic trajectories. Beyond standard Partial Least Squares Discriminant Analysis (PLS-DA) and hierarchical clustering, Orthogonal PLS-DA (OPLS-DA) isolated specific group divergences. A 200-iteration permutation test was performed to evaluate model robustness and reduce concerns regarding overfitting. The differential metabolites were subsequently subjected to KEGG pathway enrichment analysis.

Differential metabolites used for heatmap visualization were defined by the comparison of Ang II + Baicalin versus Ang II using predefined criteria, including VIP > 1.0, absolute log2 fold change > 0.58, and FDR-adjusted q < 0.05. The heatmap displays the top-ranked differential metabolites according to FDR-adjusted q values. KEGG pathway enrichment was performed using differential metabolites identified from the Ang II + Baicalin versus Ang II comparison. When directionality was evaluated, metabolites increased after baicalin treatment relative to Ang II were interpreted as baicalin-restored metabolic features, whereas metabolites decreased after baicalin treatment were interpreted as baicalin-suppressed metabolic features. The full differential metabolite list, including metabolite name, VIP score, log2 fold change, raw P-value, FDR-adjusted q-value, and KEGG pathway annotation, is provided in Supplementary Table S2.

To validate specific pathway signals, targeted LC-MS/MS was used to precisely quantify the absolute short-chain fatty acid (SCFA) concentrations. Caecal samples (50 mg) were homogenised in cold methanol/water (4:1, v/v) containing isotope-labelled internal standards, sonicated (30 min at 4 °C), and centrifuged (12,000 rpm for 15 min). Derivatization with 2-Nitrophenylhydrazine (2-NPH) hydrochloride maximised the ionisation efficiency. Chromatographic separation was performed using a UPLC system coupled to a QTRAP 6500+ mass spectrometer (SCIEX). Data acquisition was performed using the Multiple Reaction Monitoring (MRM) mode. Standard curves were used to calculate absolute concentrations, which were reported as μmol/g caecal content.

### 2.6 *In vivo* systemic intestinal permeability and endotoxemia assays

Fluorescein isothiocyanate (FITC)-dextran tracers were used to evaluate systemic barrier integrity *in vivo*. Following a 4-h fast, mice received oral gavage of either 4 kDa or 70 kDa FITC-dextran (600 mg/kg, Sigma-Aldrich) to differentially assess paracellular and macromolecular leak pathways. A microplate reader (SpectraMax iD3) was used to quantify serum fluorescence (excitation 490 nm, emission 520 nm) 4 h post-gavage.

A chromogenic Limulus Amebocyte Lysate (LAL) assay (Cusabio) was used to quantify plasma Lipopolysaccharide (LPS) levels, indexing systemic endotoxaemia. To determine the luminal free endotoxin burden, precisely weighed caecal contents were homogenised in endotoxin-free phosphate-buffered saline (PBS) and centrifuged (10,000 × *g*, 15 min, 4 °C). The identical LAL assay quantified free LPS in the supernatant and normalised it to the initial caecal content weight (EU/g).

### 2.7 Flow cytometry analysis

Mechanical disruption of the spleen and mesenteric lymph nodes (MLNs) using 70-μm nylon strainers yielded single-cell suspensions. RBC Lysis Buffer (Solarbio) was used to clear erythrocytes from the splenic samples. To eliminate non-specific binding, cells (1 × 10^6) were incubated with Fc Block (anti-CD16/32, BioLegend). Fixable Viability Dye eFluor™ 780 (eBioscience) was used to exclude necrotic cells. Surface labelling was performed using fluorophore-conjugated antibodies targeting CD3 (FITC) and CD4 (APC). Following fixation and permeabilisation using the Foxp3/Transcription Factor Staining Buffer Set (eBioscience), the cells underwent intracellular staining for Foxp3 (PE) and RORγt (BV421). A CytoFLEX flow cytometer was used to acquire the data, and FlowJo v10.8.1 software was used for downstream analysis (Arpaia et al., 2013).

### 2.8 Histology and immunofluorescence staining

Thoracic aortas and colon tissues were fixed in 4% paraformaldehyde, embedded in paraffin, and sectioned (5 μm). Haematoxylin and eosin (H&E) staining was used to capture the general morphology. For immunofluorescence, the sections were subjected to antigen retrieval using citrate buffer (pH 6.0) and blocking with 5% donkey serum. The primary antibodies, anti-α-SMA (1:200, Servicebio) and anti-Foxp3 (1:100, eBioscience), were incubated overnight at 4 °C. Subsequent incubation with Alexa Fluor 488- and 594-conjugated secondary antibodies (1:500) was used to visualise the targets. The nuclei were counterstained with DAPI. High-resolution images were acquired using a Leica Stellaris 5 confocal microscope.

### 2.9 Western blotting

RIPA buffer supplemented with 1 mM PMSF and a phosphatase inhibitor cocktail (Roche) was used to extract total protein from colonic tissues and cultured cells. A BCA assay was used to quantify the protein yield. Equal protein aliquots (30 μg) were resolved via 10% SDS-PAGE before being transferred to PVDF membranes. Following 5% BSA blockade, membranes were incubated overnight at 4 °C with primary antibodies: ZO-1, Occludin, ERK1/2, p-ERK1/2 (Thr202/Tyr204), and β-actin. An ECL Chemiluminescence Kit (Tanon) was used to visualise the bands, and densitometry was quantified using ImageJ.

### 2.10 Primary cell isolation and Co-culture system

Enzymatic digestion (2 mg/mL Collagenase Type II, 40 min, 37 °C) was used to isolate primary vascular smooth muscle cells (VSMCs) from murine thoracic aortas. Cultures were expanded in DMEM supplemented with 20% FBS; passages 3–5 were used for all experiments. A CD4^+^ T Cell Isolation Kit (Miltenyi Biotec) was used to purify naïve CD4^+^ T cells from the spleens. To generate butyrate-licenced Tregs, naïve T cells underwent 72-h activation via plate-bound anti-CD3 (5 μg/mL) and soluble anti-CD28 (2 μg/mL), supplemented with TGF-β1 (5 ng/mL), IL-2 (10 ng/mL), and Sodium Butyrate (0.5 mM).

Co-culture assays were performed using 24-well Transwell plates (0.4 μm pore size). VSMCs occupied the lower chamber, and differentiated Tregs or Th17 cells populated the upper inserts at a 1:5 (VSMC: T cells) ratio. Targeted neutralisation assays were used to map paracrine effector molecules by supplementing the lower chamber with antibodies that neutralise IL-10 (10 μg/mL) or TGF-β (10 μg/mL), alongside an Isotype IgG control.

Cell viability was measured using the Cell Counting Kit-8 (CCK-8) at 450 nm. Flow cytometric quantification using the EdU Cell Proliferation Kit was used to assess the fraction of cells actively synthesising DNA (EdU+) in the S phase.

### 2.11 Statistical analysis

Data are presented as mean ± Standard Error of the Mean (SEM). The Shapiro-Wilk test was used to verify data normality. For cross-sectional physiological and histological endpoints, multiple group comparisons were performed using One-way Analysis of Variance (ANOVA) followed by Tukey’s *post hoc* test. For longitudinal blood pressure measurements (e.g., weekly SBP tracking), a repeated-measures two-way ANOVA was employed to assess time-by-treatment interactions. Multi-omics analyses, including 16S rRNA sequencing and untargeted metabolomics, were considered exploratory and hypothesis-generating analyses. Targeted SCFA quantification, intestinal permeability assays, LPS measurements, flow cytometry, histology, Western blotting, and *in vitro* co-culture experiments were treated as confirmatory analyses supporting the proposed mechanistic axis.

Because this study was designed as a mechanism-oriented preclinical investigation, non-omics endpoints were interpreted within predefined biological domains rather than as independent discovery screens. For endpoint-level analyses, Tukey’s *post hoc* correction was applied within each outcome after ANOVA. For high-dimensional multi-omics datasets, including 16S rRNA sequencing and untargeted metabolomics, raw P-values were adjusted using the Benjamini-Hochberg false discovery rate (FDR) procedure. Differential taxa or metabolites were considered statistically significant when the FDR-adjusted P-value (q-value) was <0.05. Heatmaps displaying these features used row Z-scores calculated by standardizing the relative abundance or intensity of each feature across biological replicates. GraphPad Prism 9.5.0 and R software (v4.2.0) were used for statistical testing and visualization. Statistical significance for non-omics data was set at P < 0.05.

## 3 Results

### 3.1 Baicalin attenuates Ang II-induced hypertension and endotoxemia in a microbiota-associated manner

Before evaluating therapeutic efficacy in the Ang II-induced hypertension model, we performed a preliminary safety assessment of the selected doses in healthy mice. After 28 days of treatment, H&E staining of the heart, liver, and kidney showed preserved tissue architecture without discernible inflammatory infiltration, necrosis, or structural injury in mice receiving baicalin at 50 mg/kg/day or NaB at 200 mg/kg/day (Supplementary Figure S1A). These findings suggest that the selected doses did not induce obvious major-organ histopathological injury under the present experimental conditions.

**FIGURE 1:**
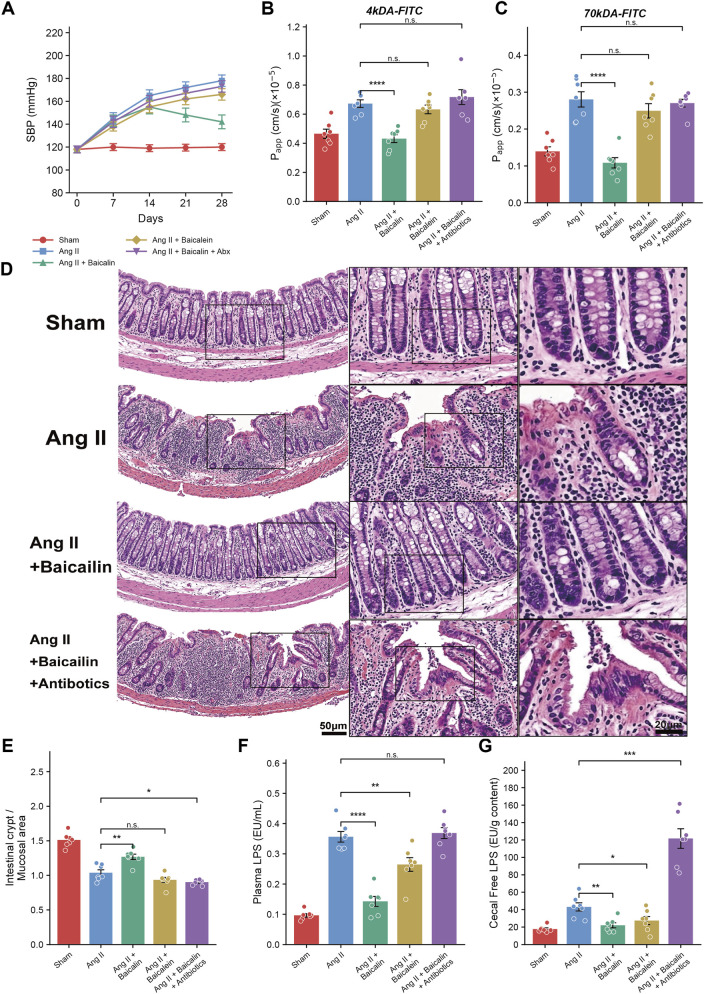
Baicalin attenuates Ang II-induced hypertension and restores systemic intestinal barrier integrity via a microbiota-dependent mechanism. **(A)** Longitudinal monitoring of systolic blood pressure (SBP) over 28 days via tail-cuff plethysmography. Mice were administered Baicalin (oral), Baicalein (i.p.), or Baicalin + Antibiotics alongside Ang II infusion. **(B,C)** Assessment of *in vivo* systemic intestinal permeability. Serum fluorescence concentrations were measured 4 h after oral gavage of 4 kDa FITC-dextran **(B)** or 70 kDa FITC-dextran **(C)**. **(D)** Representative Hematoxylin and Eosin (H&E) staining of colonic sections. Scale bars: 50 μm (left) and 20 μm (right). **(E)** Histological quantification of the ratio of intestinal crypt depth to mucosal area. **(F)** Quantification of plasma lipopolysaccharide (LPS) levels by ELISA. **(G)** Quantification of free LPS concentrations within the cecal contents. Data are presented as mean ± SEM (n = 7 per group). For longitudinal SBP data in **(A)**, repeated-measures two-way ANOVA was used to evaluate time, treatment, and time-by-treatment interaction effects. For endpoint measurements in **(B,C,E–G)**, one-way ANOVA followed by Tukey’s *post hoc* test was used. Brackets indicate the specific pairwise comparisons shown in each panel. *P < 0.05, **P < 0.01, ***P < 0.001, ****P < 0.0001; n.s., not significant.

Chronic Ang II infusion drove systolic blood pressure (SBP) to a pathological plateau, approaching 170 mmHg. Oral Baicalin intercepted this trajectory, stabilising the SBP at approximately 135 mmHg. This protective phenotype was strongly attenuated by antibiotic-induced microbiota depletion, as the blood pressure profile in the Ang II + Baicalin + Antibiotics group closely resembled that of the untreated Ang II cohort (Figure 1A). These findings support a major contribution of the gut microbiota to the observed protective effects of oral baicalin under the present experimental conditions. Parallel assessments of diastolic blood pressure (DBP) and mean arterial pressure (MAP) confirmed comprehensive haemodynamic rescue (Supplementary Figures S1B,C). This haemodynamic rescue is paralleled by structural preservation at the intestinal interface. Ang II compromised overall epithelial integrity, driving a >2-fold increase in the systemic transmucosal flux of both 4 kDa and 70 kDa FITC-dextran tracers.

Baicalin reinforced this barrier, restricting macromolecular leakage to near-physiological rates (Figures 1B,C). Histological assessment corroborated these functional assays; baicalin prevented Ang II-induced crypt atrophy and mucosal erosion (Figures 1D,E). We subsequently quantified the systemic translocation of luminal endotoxin. Plasma LPS levels surged to ∼0.35 EU/mL under Ang II stress, whereas Baicalin administration suppressed endotoxaemia to baseline levels (Figure 1F). Parallel quantification of free LPS within caecal contents revealed Ang II-driven luminal accumulation, which was effectively attenuated by BA (Figure 1G). Antibiotic depletion was associated with increased caecal free LPS levels, potentially reflecting bacterial lysis and altered luminal endotoxin handling.

Baicalin Reconstructs the Gut Microbial Architecture and Restores a Butyrate-Associated Microbial Signature.

**FIGURE 2:**
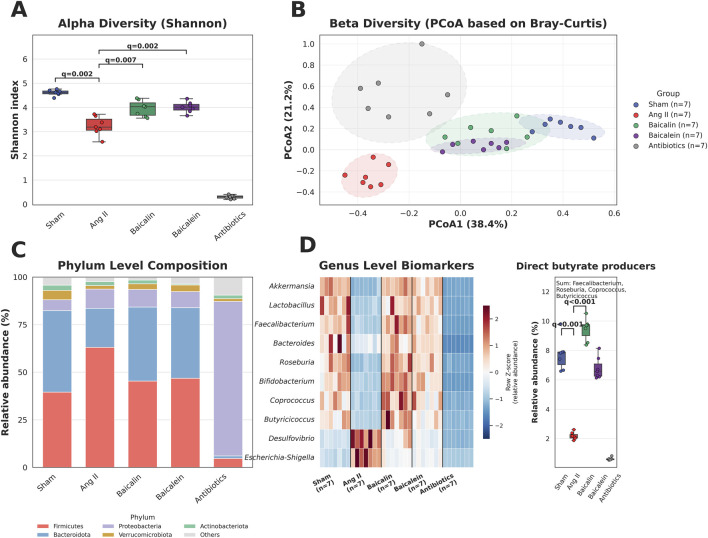
Baicalin reconstructs the gut microbial architecture and restores a butyrate-associated microbial signature. **(A)** Alpha diversity of the cecal microbiota as measured by the Shannon index. **(B)** Beta diversity visualization using Principal Coordinate Analysis (PCoA) based on Bray-Curtis dissimilarity. **(C)** Taxonomic composition at the phylum level. Stacked bars represent the relative abundance of *Firmicutes*, *Bacteroidetes*, *Proteobacteria*, *Verrucomicrobiota*, *Actinobacteriota*, and other phyla. **(D)** Heatmap of differentially abundant genera identified by LEfSe using predefined criteria (Kruskal-Wallis alpha = 0.05, log10 LDA score >3.0, and FDR-adjusted q < 0.05). The color scale represents the row Z-score of standardized relative abundance. The rightmost panel quantifies the summed relative abundance of putative butyrate-associated genera, including Faecalibacterium, Roseburia, Coprococcus, and Butyricicoccus. q-values were calculated using Kruskal-Wallis testing followed by FDR correction. Data are shown for n = 7 per group.

16S rRNA gene sequencing revealed a profound dysbiotic shift following Ang II infusion. Alpha diversity contracted sharply (Shannon index: ∼4.5 to ∼3.0, *P* < 0.001). Oral Baicalin counteracted this ecological erosion, restoring community richness to ∼4.0. The antibiotic cocktail induced pseudo-sterility (Shannon index < 0.5), validating the depletion model (Figure 2A). Beta diversity analysis corroborated this restoration trajectory. Principal Coordinate Analysis (PCoA) demonstrated that Ang II drove the microbial centroid distinctively away from the sham cluster, whereas baicalin compelled migration back toward the baseline phenotype (Figure 2B). Taxonomic profiling revealed structural remodelling at the phylum level. Ang II expanded *Firmicutes* at the expense of *Bacteroidetes*, elevating the F/B ratio. Baicalin recalibrated this equilibrium by suppressing the *Firmicutes* bloom and normalising the community composition (Figure 2C). Genus-level LEfSe analysis identified differentially abundant microbial biomarkers using predefined criteria, including a Kruskal-Wallis alpha value of 0.05, a log10 LDA score threshold >3.0, and FDR-adjusted q < 0.05. Figure 2D displays the top-ranked genus-level biomarkers derived from this predefined output. Baicalin markedly enriched Akkermansia and *Lactobacillus*, which are more appropriately interpreted as SCFA-supporting or cross-feeding-associated genera rather than direct butyrate producers. To further examine the microbial basis of increased luminal butyrate, we quantified a predefined set of canonical butyrate-associated genera, including Faecalibacterium, Roseburia, Coprococcus, and Butyricicoccus (Figure 2D). Their summed relative abundance was depleted by Ang II and restored by baicalin treatment, supporting a cross-feeding ecological model rather than a single-genus mechanism.

### 3.2 Baicalin reprograms gut metabolism and elevates luminal butyrate levels

**FIGURE 3:**
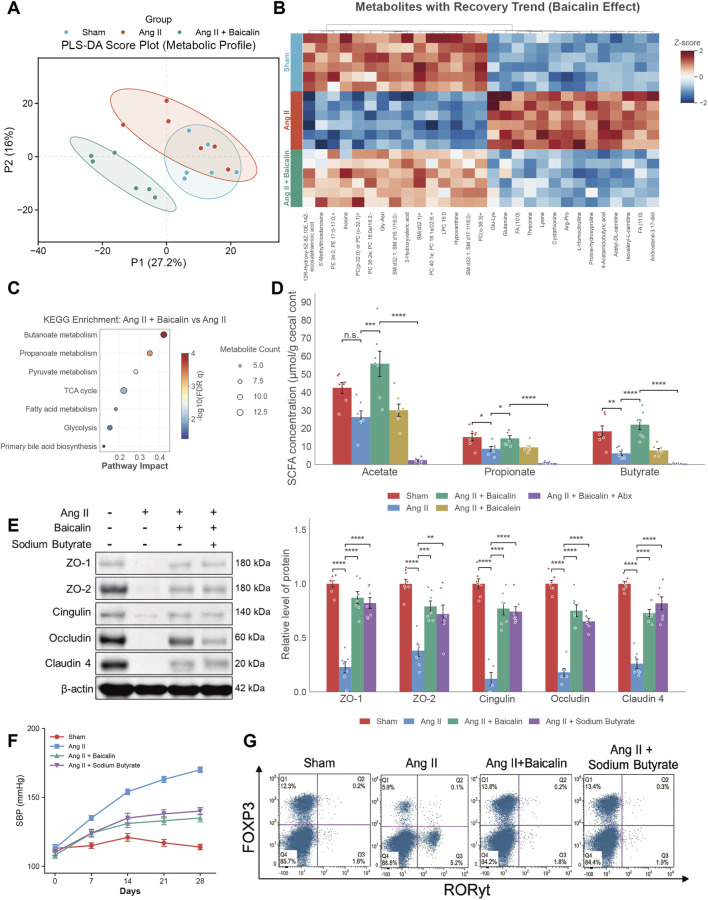
Baicalin reprograms global gut metabolism and restores luminal butyrate to protect the intestinal barrier. **(A)** Partial Least Squares Discriminant Analysis (PLS-DA) score plot derived from untargeted metabolomic profiling of cecal contents. **(B)** Hierarchical clustering heatmap of differential metabolites selected from the Ang II + Baicalin versus Ang II comparison according to predefined multivariate and FDR-adjusted criteria. The color scale represents row Z-scores of metabolite intensity. **(C)** KEGG pathway enrichment analysis of differential metabolites identified from the Ang II + Baicalin versus Ang II comparison. The pathways depicted should be interpreted as baicalin-associated metabolic restoration relative to the Ang II model group rather than as non-directional global enrichment. **(D)** Targeted absolute quantification of short-chain fatty acids (SCFAs), including Acetate, Propionate, and Butyrate, in cecal contents using LC-MS/MS. **(E)** Representative Western blot analysis and densitometric quantification of tight junction proteins (ZO-1, ZO-2, Cingulin, Occludin, and Claudin-4) in colonic tissues. Sodium Butyrate (NaB) was administered to validate the metabolic mechanism. Protein levels were normalized to β-actin. **(F)** Longitudinal SBP measurements following exogenous NaB supplementation. **(G)** Representative flow cytometry plots of splenic CD4^+^ T cells gated for Foxp3 (y-axis) and RORγt (x-axis). Data are presented as mean ± SEM (n = 7 per group). Brackets indicate the exact pairwise comparisons shown in each panel. For longitudinal SBP data in **(F)**, repeated-measures two-way ANOVA followed by Tukey’s *post hoc* test was used. For endpoint measurements, one-way ANOVA followed by Tukey’s *post hoc* test was used. *P < 0.05, **P < 0.01, ***P < 0.001, ****P < 0.0001; n.s., not significant.

Untargeted metabolomic profiling delineated global metabolic reprogramming in the caecal microenvironment. PLS-DA segregated the Ang II cohort from the baicalin-treated cohort, indicating a distinct metabolic shift induced by baicalin treatment (Figures 3A,B). Differential metabolites displayed in the heatmap were selected from the Ang II + Baicalin versus Ang II comparison according to predefined multivariate and FDR-adjusted criteria. OPLS-DA further validated this treatment-associated metabolic divergence (R^2^Y = 0.982, Q^2^ = 0.935), while a 200-permutation test conclusively eliminated overfitting concerns, yielding a negative Q^2^ intercept (Supplementary Figures S1D,E).

KEGG enrichment analysis was therefore interpreted as pathway mapping of metabolites altered in Ang II + Baicalin versus Ang II, rather than as a non-directional global pathway list. This analysis highlighted butanoate and propanoate metabolism as major metabolic pathways associated with baicalin-mediated metabolic restoration relative to the Ang II model group (Figure 3C). Targeted LC-MS/MS was used to quantify the absolute SCFA concentrations to validate these pathway signals. Ang II infusion severely depleted absolute caecal butyrate levels. Conversely, oral Baicalin robustly restored the butyrate pool, alongside an elevation in acetate (Figure 3D). To isolate the biological efficacy of this specific metabolic shift, we administered exogenous Sodium Butyrate (NaB). Western blotting of colonic lysates revealed that Ang II downregulated critical tight junction proteins, thereby compromising the epithelial barrier. NaB supplementation rescued this structural deficit, restoring protein expression to levels statistically indistinguishable from those in the baicalin-treated cohort (Figure 3E). This mucosal reinforcement directly translates into systemic therapeutic benefits. NaB attenuated the Ang II-dependent SBP increase (Figure 3F) and recapitulated the baicalin-driven expansion of the splenic Foxp3+ Treg population (Figure 3G).

### 3.3 Baicalin corrects systemic Th17/Treg imbalance via a gut-lymphatic priming mechanism

**FIGURE 4:**
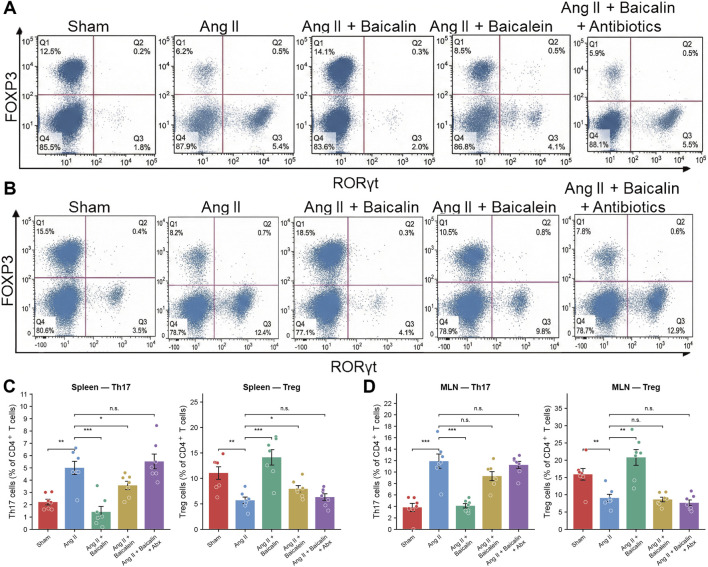
Baicalin corrects systemic Th17/Treg imbalance via a gut-lymphatic priming mechanism. **(A,B)** Representative flow cytometry plots of CD4^+^ T cells isolated from the spleen **(A)** and mesenteric lymph nodes (MLNs) **(B)**. Cells were gated on CD3^+^CD4^+^ and analyzed for intracellular Foxp3 and RORγt expression. **(C)** Quantification of Th17 and Treg frequencies in the spleen. **(D)** Quantification of Th17 (RORγt+) and Treg (Foxp3+) cell frequencies in the MLNs. Data are presented as mean ± SEM (n = 7 per group). Brackets indicate the exact pairwise comparisons shown in each panel. One-way ANOVA followed by Tukey’s *post hoc* test was used. *P < 0.05, **P < 0.01, ***P < 0.001, ****P < 0.0001; n.s., not significant.

The spleen acts as a barometer for systemic immune tone; therefore, we profiled splenic CD4^+^ T cell polarisation. Ang II infusion precipitated a disruption of peripheral immune balance, contracting the Foxp3+ Treg pool from a baseline of ∼12.5% to ∼6.2%, while driving RORγt+ Th17 expansion to ∼5.4% (Figures 4A,C). Oral Baicalin interrupted this inflammatory drift. The intervention restored the Treg population to ∼14.1% and suppressed the Th17 frequency to near-baseline levels (∼2.0%). Antibiotic-induced sterility markedly weakened this rescue, locking the immune phenotype in a pro-inflammatory state (Treg: ∼5.9%, Th17: ∼5.5%). We interrogated the mesenteric lymph nodes (MLNs) to pinpoint the anatomical origin of immune restoration. Therapeutic dependency on the administration route was highly evident. Intraperitoneal injection of the aglycone baicalein elicited marginal recovery (Treg: ∼10.5%). Oral Baicalin, however, drove a robust immunological rebound, boosting MLN Treg frequencies to ∼18.5%, exceeding that of the sham control (Figures 4B,D). Antibiotic co-treatment obliterated this preferential expansion in the gut-draining lymphatics (Treg: ∼7.8%), indicating that baicalin licences regulatory T cells locally within the gut-associated lymphoid tissue prior to systemic distribution.

### 3.4 Baicalin increases adventitial Foxp3+ regulatory immune-cell accumulation and attenuates vascular remodelling

**FIGURE 5:**
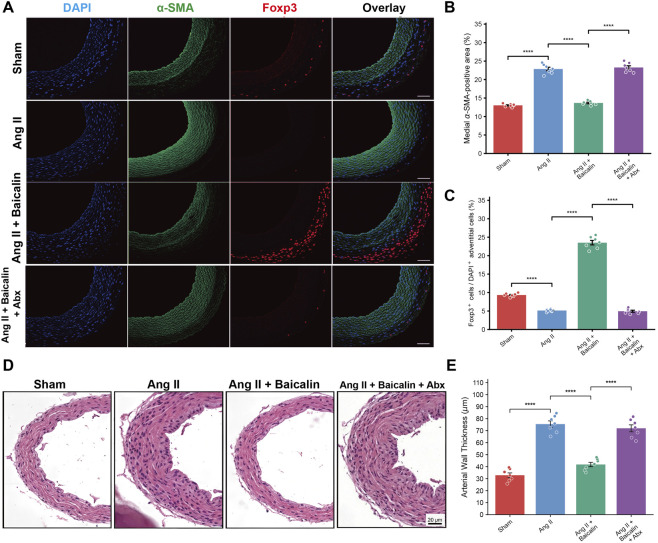
Baicalin increases adventitial Foxp3+ regulatory immune-cell accumulation and attenuates vascular remodelling. **(A)** Representative immunofluorescence images of thoracic aorta sections stained for α-SMA (green), Foxp3 (red), and DAPI (blue). Foxp3+ regulatory immune cells were mainly detected within the adventitial region, and this baicalin-associated accumulation was reduced by concurrent antibiotic (Abx) treatment. **(B)** Quantification of α-SMA-positive area percentage in the tunica media. **(C)** Quantification of Foxp3+ cell frequency in the aortic adventitia. **(D)** Representative H&E staining of thoracic aorta cross-sections. Scale bar: 20 μm. **(E)** Morphometric analysis of arterial wall thickness. Data are presented as mean ± SEM (n = 7 per group). Brackets indicate the exact pairwise comparisons shown in each panel. One-way ANOVA followed by Tukey’s *post hoc* test was used. *P < 0.05, **P < 0.01, ***P < 0.001, ****P < 0.0001; n.s., not significant.

Baicalin treatment was accompanied by increased Foxp3+ regulatory immune-cell accumulation within the aortic adventitial niche (Figure 5A). Quantification confirmed that the frequency of adventitial Foxp3+ cells was markedly increased in the baicalin-treated group compared with the Ang II group (Figure 5C **)**. Coinciding with this local immune shift, baicalin attenuated Ang II-induced vascular remodelling. Histological morphometry demonstrated reduced arterial wall thickening in baicalin-treated mice compared with Ang II-treated mice (Figures 5D,E). Molecular interrogation further showed that Ang II-induced α-SMA overexpression, a marker associated with maladaptive smooth muscle remodelling, was reduced after baicalin treatment (Figure 5B). These findings suggest that baicalin-associated regulatory immune remodelling in the adventitia is linked to improved vascular structural outcomes.

### 3.5 Butyrate-licensed tregs attenuate Ang II-induced VSMC proliferative responses via the MAPK/ERK axis

**FIGURE 6:**
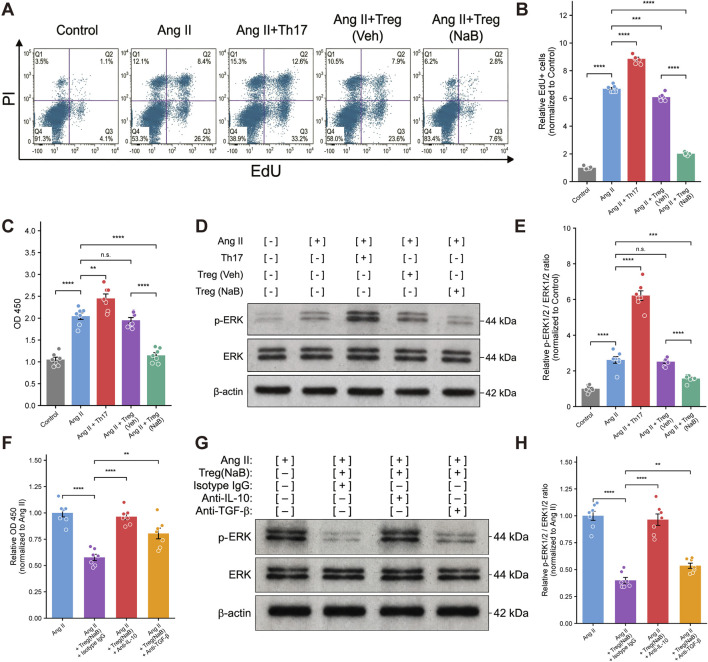
Butyrate-licensed Tregs attenuate Ang II-induced VSMC proliferative responses via the MAPK/ERK axis. **(A)** Representative flow cytometry plots of EdU/PI staining in primary vascular smooth muscle cells (VSMCs). VSMCs were treated with Ang II (100 nM) and co-cultured with Th17 cells, unconditioned Tregs (Treg (Veh)), or Tregs differentiated in the presence of sodium butyrate (Treg (NaB)). **(B)** Quantification of the VSMC proliferation rate, calculated as EdU+ cells (Q2 + Q3). **(C)** Analysis of overall VSMC viability/proliferative activity assessed by CCK-8 assay (OD at 450 nm). **(D)** Representative Western blot analysis of phosphorylated ERK1/2 (p-ERK) and total ERK1/2 in VSMCs. β-actin served as the loading control. **(E)** Densitometric quantification of the p-ERK/ERK ratio. **(F)** Assessment of VSMC proliferation by CCK-8 assay following co-culture with NaB-licensed Tregs in the presence of neutralizing antibodies against IL-10, TGF-β, or an isotype IgG control. **(G)** Representative Western blot of p-ERK and total ERK in VSMCs subjected to the neutralization assay. **(H)** Densitometric quantification of the p-ERK/ERK ratio from the neutralization assay. Data are presented as mean ± SEM (n = 7 per group). Brackets indicate the exact pairwise comparisons shown in each panel. One-way ANOVA followed by Tukey’s *post hoc* test was used for **(B,C,E,F,H)**. *P < 0.05, **P < 0.01, ***P < 0.001, ****P < 0.0001; n.s., not significant.

Cellular assays were used to evaluate the regulatory capacity of Tregs on VSMCs. EdU incorporation was used to quantify DNA synthesis. Ang II increased the EdU + fraction of VSMCs, and this response was further enhanced during co-culture with Th17 cells. Co-culture with unconditioned vehicle-treated Tregs showed limited suppression, whereas NaB-licensed Tregs markedly reduced Ang II-induced VSMC proliferation (Figures 6A,B). The CCK-8 assay showed a broadly consistent pattern in overall cell viability/proliferative activity (Figure 6C). Molecular interrogation indicated that the MAPK/ERK pathway was involved in this immune-regulatory effect. Ang II increased ERK1/2 phosphorylation, Th17 exposure further enhanced this signal, and NaB-licensed Tregs reduced p-ERK levels compared with Ang II-treated VSMCs (Figures 6D,E).

Targeted neutralisation assays suggested that IL-10 contributed substantially to this suppressive effect. Isotype IgG did not alter the effect of NaB-licensed Tregs, whereas IL-10 blockade restored VSMC proliferative activity and reactivated ERK phosphorylation (Figures 6F–H). TGF-β blockade produced a partial intermediate reversal, suggesting that IL-10 may be a major, but not necessarily exclusive, paracrine mediator in this co-culture system.

## 4 Discussion

This study addresses the pharmacokinetic discordance in baicalin pharmacology. Despite its limited oral bioavailability, baicalin has been reported to exert vasculoprotective and antihypertensive effects in experimental models (Si and Lai, 2024). Our findings support the gut microbiota as an important contributor to the protective phenotype observed after oral baicalin administration, rather than establishing it as the exclusive mediator. Consistent with previous evidence that baicalin can modulate gut microbial ecology (Wu et al., 2019), baicalin treatment reshaped SCFA-supporting commensals, including Akkermansia and *Lactobacillus*, and restored a putative butyrate-associated microbial signature. This ecological shift was accompanied by increased luminal butyrate availability and regulatory immune remodelling in mesenteric lymph nodes (MLNs). Given the known role of butyrate in promoting Foxp3+ regulatory T cell (Treg) differentiation (Furusawa et al., 2013), these findings suggest that baicalin-associated microbial metabolic remodelling may contribute to restoration of the Th17/Treg balance. Increased Foxp3+ regulatory immune-cell accumulation in the aortic adventitia and reduced MAPK/ERK activation in VSMCs further support a gut microbiota–butyrate–regulatory immunity axis contributing to attenuation of Ang II-induced vascular remodelling.

Gut health is increasingly recognised as a contributor to vascular tone, although the translational bridge between microbial ecology and vascular remodelling requires further mechanistic validation (Yang et al., 2025). Hypertension has been associated with gut dysbiosis, including altered Firmicutes/Bacteroidetes balance and depletion of SCFA-associated microbial functions (Yang et al., 2015; Marques et al., 2017). In our study, baicalin partially restored this dysbiotic pattern and increased the relative abundance of Akkermansia muciniphila and *Lactobacillus*. Akkermansia has been implicated in metabolic homeostasis and mucosal barrier regulation (Cani et al., 2022), and specific Akkermansia-derived factors have been reported to support epithelial tight-junction integrity (Kim et al., 2024).

Importantly, the primary responders identified in our genus-level profiling, including Akkermansia and *Lactobacillus*, should not be interpreted as canonical direct butyrate producers. Rather, these taxa may contribute to mucosal stabilization and upstream SCFA-related cross-feeding by providing acetate, lactate, or other substrates that support secondary butyrate-associated microbial communities. Consistent with this interpretation, our updated genus-level analysis showed that a predefined set of putative butyrate-associated genera, including Faecalibacterium, Roseburia, Coprococcus, and Butyricicoccus, was reduced under Ang II stress and partially restored after baicalin treatment. Thus, baicalin may act as an ecological modulator that supports an upstream cross-feeding network, thereby contributing to increased luminal butyrate availability. This interpretation integrates the taxonomic data with targeted SCFA quantification while avoiding over-attribution of butyrate production to individual genera (Snelson et al., 2024).

These findings may also help explain the persistent endotoxemia observed after antibiotic-induced microbiota depletion. Systemic endotoxemia can persist despite a marked reduction in viable LPS-producing Gram-negative bacteria (Reikvam et al., 2011). In our model, paired assessment of luminal and systemic LPS suggested that broad-spectrum antibiotic treatment increased the luminal free endotoxin burden, potentially through bacterial lysis. Because Ang II stress and antibiotic exposure were both associated with impaired epithelial barrier integrity, this elevated luminal LPS pool may have facilitated systemic endotoxin translocation, thereby maintaining pathological plasma LPS levels (Richards et al., 2022). In contrast, baicalin appeared to act as an ecological modulator that reduced luminal endotoxin burden while improving epithelial barrier integrity. These findings suggest that baicalin-associated microbial and barrier remodeling may jointly contribute to reduced systemic endotoxin exposure.

Butyrate appears to be an important metabolic link connecting baicalin-associated microbial remodelling with regulatory immune responses. Previous studies have linked SCFA depletion to hypertension and immune dysregulation (Furusawa et al., 2013). In the present study, targeted SCFA quantification showed that baicalin restored luminal butyrate availability, and exogenous sodium butyrate (NaB) recapitulated several major protective features of baicalin treatment, including attenuation of blood pressure elevation and promotion of Foxp3+ regulatory immune responses. Mechanistically, butyrate has been reported to function as a histone deacetylase (HDAC) inhibitor and to promote Foxp3 expression through epigenetic regulation of the Foxp3 locus (Mann et al., 2024; Arpaia et al., 2013). Therefore, our data are consistent with a model in which baicalin-associated restoration of luminal butyrate contributes to MLN regulatory immune priming and correction of the Ang II-driven Th17/Treg imbalance. The stronger effect of oral baicalin compared with intraperitoneal baicalein further supports the importance of enteral microbiota-associated processes, although systemic exposure and microbiota-independent mechanisms cannot be fully excluded (Ma et al., 2024).

Spatial dynamics of vascular protection may also be relevant to the proposed gut-immune-vascular axis. Prevailing hypertensive paradigms often focus on endothelial dysfunction or medial hypertrophy (Guzik et al., 2024), whereas the adventitia is increasingly recognised as an immunologically active compartment of the arterial wall (Totoń-Żurańska et al., 2024). In the present study, baicalin treatment was accompanied by increased Foxp3+ regulatory immune-cell accumulation within the aortic adventitial region. This observation is consistent with an outside-in immunoregulatory mechanism, although definitive evidence of lymphocyte trafficking from gut-draining lymphoid tissues to the aorta will require lineage-tracing or lymphatic egress-blockade approaches. *In vitro* Transwell assays further suggested that NaB-conditioned Tregs can suppress Ang II-induced VSMC proliferative signalling and ERK1/2 phosphorylation without direct cell-cell contact (Nosalski and Guzik, 2017). Targeted neutralisation experiments implicated IL-10 as a major paracrine mediator, whereas TGF-β blockade produced a partial reversal (Drummond et al., 2019). Therefore, the present data support an IL-10-associated paracrine regulatory model, while additional *in vivo* studies are needed to define the relative contribution of Treg-mediated immunity compared with endothelial, smooth-muscle, and direct metabolite effects (Zmora et al., 2019).

Translation of microbiome-modulating therapies must navigate inter-individual baseline microbiota variability (Selma et al., 2020). Human responses to flavonoids exhibit extreme heterogeneity, largely dictated by specific metabotypes harbouring the enzymatic machinery required for Baicalin deglycosylation (Tang et al., 2017). Other microbial metabolites, including secondary bile acids or trimethylamine N-oxide (TMAO), might also shift under Baicalin treatment, warranting parallel investigation (Guzik et al., 2007; Loperena et al., 2018). While we mapped the multi-omics framework supporting the gut-lymphatic-adventitial axis, definitive proof of spatial homing demands sophisticated *in vivo* lineage tracing, such as Kaede photoconvertible mice or FTY720-mediated lymphatic egress blockade (Harrison et al., 2021). Similarly, isolating the precise proportional contribution of Treg-mediated cellular immunity against the direct systemic vasodilatory effects of circulating butyrate requires highly specific *in vivo* Treg depletion models, utilising anti-CD25 neutralising antibodies or Foxp3-DTR transgenic mice (Mancia et al., 2023).

While this study established a multi-omics framework supporting a gut-lymphatic-adventitial axis, several limitations warrant acknowledgement. First, although we observed coordinated Treg expansion within MLNs and increased Foxp3+ regulatory immune-cell accumulation in the aortic adventitia, definitive proof of spatial migration requires *in vivo* lineage-tracing approaches, such as Kaede photoconvertible mice, or lymphatic egress-blockade experiments using FTY720. Second, although the *in vitro* Transwell assays support a paracrine suppressive effect of butyrate-licensed Tregs on VSMCs, the *in vivo* vascular microenvironment is more complex. Future studies using anti-CD25 neutralisation, Foxp3-DTR transgenic mice, or other Treg-depletion strategies are needed to define the proportional contribution of Treg-mediated immunity relative to endothelial, smooth-muscle, and direct metabolite effects. Third, this study did not include a standard clinically relevant antihypertensive positive control, such as losartan, amlodipine, or enalapril; therefore, the relative antihypertensive potency of baicalin cannot be benchmarked against established pharmacotherapy. Fourth, we did not directly quantify plasma concentrations of baicalin or its metabolites after oral administration. Although antibiotic-induced microbiota depletion supports a major contribution of the gut microbiota to the observed protective phenotype, microbiota-independent systemic actions cannot be fully excluded. Fifth, the safety evaluation was preliminary and limited by the small sample size, short observation period, and absence of dose-escalation toxicology. Future pharmacological profiling should incorporate standard positive drug controls, pharmacokinetic assessment, GLP-grade toxicological evaluation, longer-term organ safety analysis, and ideally faecal microbiota transplantation or germ-free models to further evaluate microbial sufficiency. Finally, although tail-cuff plethysmography provided reliable longitudinal blood pressure trends under acclimation, radiotelemetry would provide more continuous and precise haemodynamic profiling in future studies.

## 5 Conclusion

This study provides preclinical evidence that baicalin attenuates Ang II-induced hypertension and vascular remodeling through a gut microbiota-associated immunometabolic mechanism involving intestinal barrier restoration, increased luminal butyrate availability, regulatory immune priming, and suppression of vascular inflammatory remodeling. These findings support the gut microbiota as an important and potentially druggable interface for hypertension-associated vascular remodeling. Rather than establishing baicalin as a verified antihypertensive therapy, our data identify baicalin as a promising microbiome-reprogramming candidate that warrants further validation using standard positive drug controls, pharmacokinetic profiling, microbial sufficiency experiments, and long-term safety assessment.

## Data Availability

The data presented in the study are deposited in the figshare repository, URL: https://doi.org/10.6084/m9.figshare.32145166

## Supplementary material

SUPPLEMENTARY FIGURE S1 Preliminary organ histological safety evaluation, additional hemodynamic parameters, and metabolomic model validation. Healthy mice were treated with vehicle, baicalin at 50 mg/kg/day, or sodium butyrate (NaB) at 200 mg/kg/day for 28 days. **(A)** Representative H&E staining images of heart, liver, and kidney tissues at the endpoint. Scale bar = 100 μm. **(B)** Longitudinal monitoring of Diastolic Blood Pressure (DBP) over 28 days via tail-cuff plethysmography. **(C)** Longitudinal monitoring of Mean Arterial Pressure (MAP) over 28 days. Data in A and B are presented as mean ± SEM (n = 7). **(D)** Orthogonal Partial Least Squares Discriminant Analysis (OPLS-DA) score plot mapping the metabolic divergence between the Ang II and Ang II + Baicalin cohorts. Shaded ellipses represent the 95% confidence intervals. **(E)** A 200-iteration permutation test validating the OPLS-DA model. The negative y-intercept of the Q^2^ regression line confirms the absence of model overfitting.
