# Supplementary figures and images for: Gut microbiota-derived butyrate contributes to baicalin-induced attenuation of hypertensive vascular remodeling via adventitial immunity
Source: Front Pharmacol. 2026 Jul 3;17:1835174. doi: 10.3389/fphar.2026.1835174 (PMC13375513; doi:10.3389/fphar.2026.1835174)

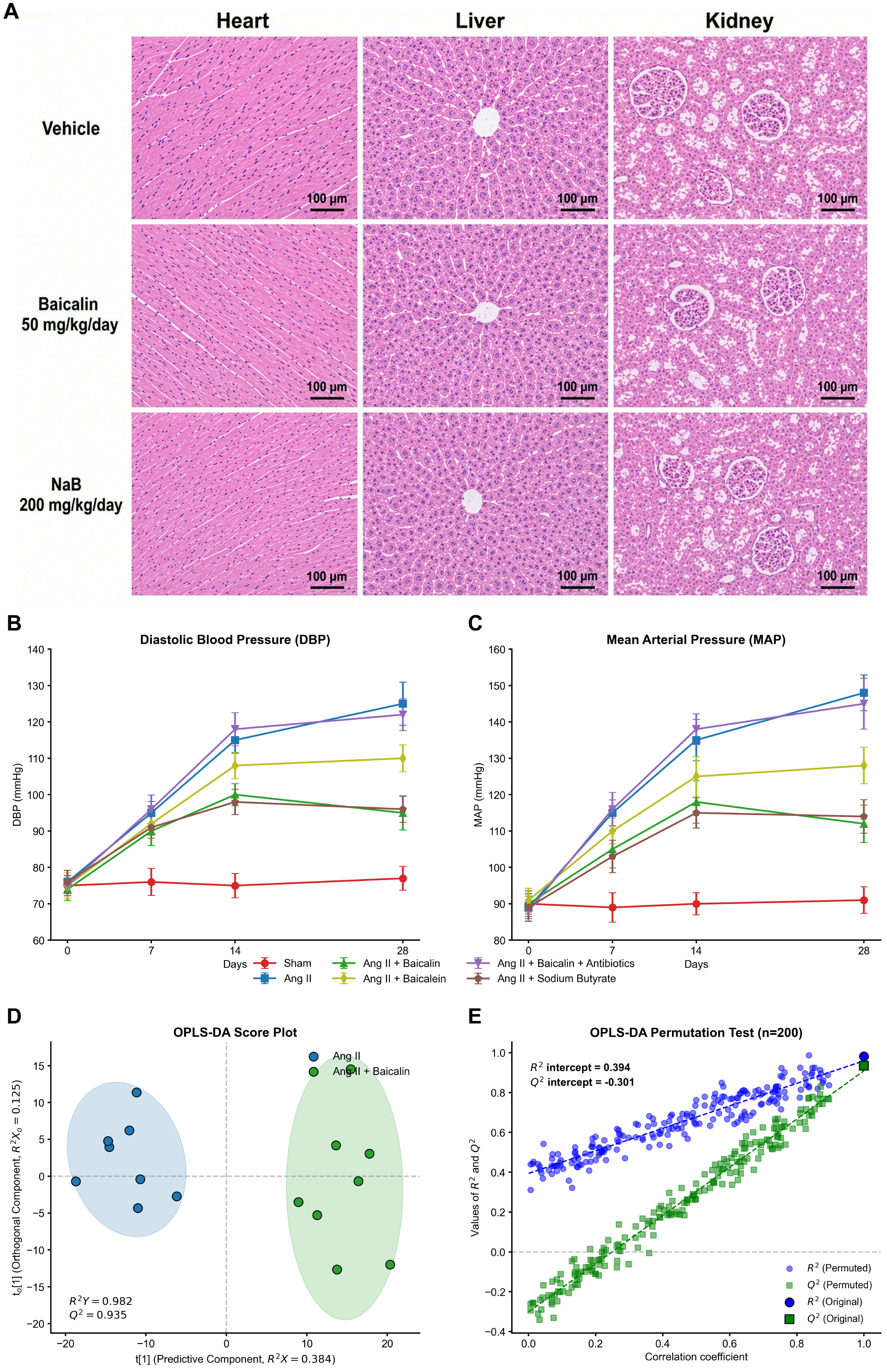

Supplement: Supplementary file 2 [file Image1.jpeg]
